# Plausibility of a Neural Network Classifier-Based Neuroprosthesis for Depression Detection via Laughter Records

**DOI:** 10.3389/fnins.2019.00267

**Published:** 2019-03-21

**Authors:** Jorge Navarro, Mercedes Fernández Rosell, Angel Castellanos, Raquel del Moral, Rafael Lahoz-Beltra, Pedro C. Marijuán

**Affiliations:** ^1^Aragon Institute of Health Science (IACS), Zaragoza, Spain; ^2^Aragon Health Research Institute (IIS Aragón), Zaragoza, Spain; ^3^Department of Biodiversity, Ecology, Evolution (Biomathematics), Faculty of Biological Sciences, Complutense University of Madrid, Madrid, Spain; ^4^Department of Applied Mathematics, Universidad Politécnica de Madrid, Madrid, Spain

**Keywords:** neuroprosthesis, neural network classifiers, laughter sound structures, depression detection, neuropsychiatry

## Abstract

The present work explores the diagnostic performance for depression of neural network classifiers analyzing the sound structures of laughter as registered from clinical patients and healthy controls. The main methodological novelty of this work is that simple sound variables of laughter are used as inputs, instead of electrophysiological signals or local field potentials (LFPs) or spoken language utterances, which are the usual protocols up-to-date. In the present study, involving 934 laughs from 30 patients and 20 controls, four different neural networks models were tested for sensitivity analysis, and were additionally trained for depression detection. Some elementary sound variables were extracted from the records: timing, fundamental frequency mean, first three formants, average power, and the Shannon-Wiener entropy. In the results obtained, two of the neural networks show a diagnostic discrimination capability of 93.02 and 91.15% respectively, while the third and fourth ones have an 87.96 and 82.40% percentage of success. Remarkably, entropy turns out to be a fundamental variable to distinguish between patients and controls, and this is a significant factor which becomes essential to understand the deep neurocognitive relationships between laughter and depression. In biomedical terms, our neural network classifier-based neuroprosthesis opens up the possibility of applying the same methodology to other mental-health and neuropsychiatric pathologies. Indeed, exploring the application of laughter in the early detection and prognosis of Alzheimer and Parkinson would represent an enticing possibility, both from the biomedical and the computational points of view.

## Introduction: the Potential Relevance of Laughter in Depression Detection

**FIGURE 1:**
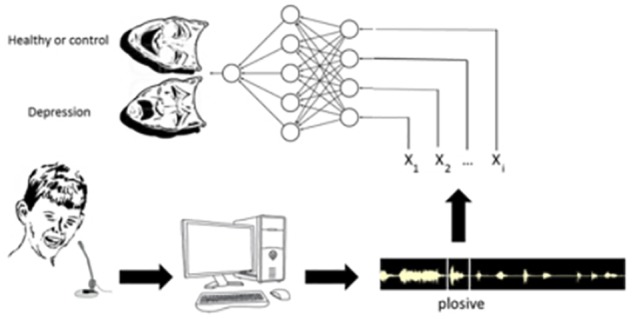
Sketch showing the main steps of the experimental protocol. First of all, laughter is recorded in patients affected by depression and in controls (healthy subjects). Then, the sonograms corresponding to each laugh are broken down into plosives. Further, we extract from each plosive the values of the sound variables X_1_, X_2_, … X_n_ that will be the input of the different neural networks. Finally, once each neural network is trained, it will be included in the neuroprosthesis.

The application of neuroprostheses in neuropsychiatry has been traditionally focused on the *treatment* of physiological disorders, e.g., control of epileptic seizures, augmenting or recovering motor function and cognitive function, treatment of anxiety, major depression, etc; all of this being done mostly via neurostimulation procedures (Moxon and Foffani, 2015; Costa E Silva and Ewbank Steffen, 2017); for instance, neuroprostheses based on EEG reading and neurofeedback (Hardt and Kamiya, 1978). However, there is an absence of studies in which neuroprostheses are applied to the *diagnosis* of mental disorders, such as depression, Parkinson’s, bipolar disorder, etc. Filling in that diagnostic gap is the main goal of the present work. An important novelty of our work is the use as inputs of sound variables coming from sonograms of previously recorded laughter of depression patients and of healthy controls (Figure 1). Other related works have already approached the detection of depression by means of speech analysis (Low et al., 2010; Alghowinem et al., 2013) or via the correlation of different audiovisual behaviors (Sturim et al., 2011; Scherer et al., 2013). Actually a new field based on automatic analysis and machine learning methods is emerging devoted to the general recognition of depression, mood, and emotion (Valstar et al., 2016). In our case, like in those works, the goal is to go beyond the traditional psychometric protocol to approach depression and other neuropsychiatric disorders by exclusively relying on use of questionnaires in the clinic (Nair et al., 1999; Chattopadhyay et al., 2012; Mukherjee et al., 2014). In the present work we explore the diagnostic performance of a powerful communicative signal, laughter, which is emitted both spontaneously and involuntarily – and the informational content of which is scarcely understood yet. We analyze its fundamental sound variables and its diagnostic performance by means of neural network classifiers, and we also summarily compare our results with some of the works previously cited relying on speech analysis.

### Neurocomputational Challenges of Laughter

Why this diagnostic use of laughter? Laughter is in itself an important social signal endowed with deep informational content. It is an interpersonal communicative signal that is emitted spontaneously in response to a very large variety of stimuli. Although laughter ordinarily is related to social interactions and to communication through the playful use of language, many other visual, tactile, physical, chemical, environmental influences, and physiological anomalies may elicit it: from tickling and physical play, to humorous – cartoons, to imitative laughter, “schadenfreude” occasions, neuropathological conditions, NO_2_ and other anesthetic gasses, and so on (Poeck, 1985; Morreall, 1987; Provine, 2000; Ariniello, 2001; Martin, 2002; Weems, 2014). The social occurrence of laugher becomes preferentially related to a variety of *bonding dynamics* in between individuals (Marijuán and Navarro, 2011; del Moral et al., 2014; Navarro et al., 2016a,b). Thereafter, laughter appears in a wide variety of interpersonal relationships, very often triggered by linguistic exchanges, where it punctuates the meanings and behavioral implications and establishes a genuine emotional evaluation of the interrelationships. Laughter contains *valence*, as different relational contexts generate different kinds of laughter; and these differences are recognizable in the aggregate as well as in the different performances of each individual (Devillers and Vidrascu, 2007; Szameitat et al., 2009). Therefore, laughter is in itself a sophisticate, communicative social-signal loaded with highly complex relational contents. In abstract terms, the main informational “stuff” of laughter seems to relate to automatic problem-solving (Hurley et al., 2011; Marijuán and Navarro, 2011; del Moral et al., 2014; Navarro et al., 2016b), marking the occurrence of a spontaneous positive solution to the different “incongruence” problems found in the ongoing relational environment. Incongruence should be understood in an ample way, including self-congratulation, superiority, tension relief, cortical “debugging” of errors, and lexical incongruence itself. Laughter spontaneously punctuates and evaluates the ongoing social relationships around the subject. If the social relationships of the individual are disturbed (i.e., by a neuropsychiatric affection such as depression), presumably this disturbance would show up in the sound structures of his/her laughter. Actually, the complexity of the emitted sound structures of laughter appears to be quite remarkable, almost comparable to articulate language – as we will see below, it also contains the equivalent of “sentences,” “words,” and “syllables” (Bachorowski and Owren, 2002; Urbain et al., 2013). The fine analysis of all the sound variables of laughter searching for systemic differences reflecting the underlying mental states becomes an interesting – and worthwhile – neurocomputational challenge. As the authors have already realized in previous works (Navarro et al., 2014, 2016a,b), statistical methods such as discriminant analysis and binary decision trees have been useful in the detection of systematic differences between depression patients and healthy controls in their responses to the same humorous stimuli.

### The Sound Structures of Laughter

That laughter as an acoustic signal has so powerful social, cognitive, and emotional effects both on emitters and receivers is in itself an outstanding fact (Bachorowski and Owren, 2008). Seemingly, laughter’s acoustic signature has been thoroughly searched out; yet it purports a series of intriguing contents. Its structure may be described as follows (Urbain et al., 2013): firstly, it is composed of separate *episodes* (or “sentences”) that enclose some *bouts* (or “words”) which are themselves consisting of relatively large exhalation parts punctuated by brief inhalations, and the exhalation parts are in their turn containing a certain number of calls or *plosives* (pulses, or “syllables”). Numerous sound variables may be distinguished, among them (following Navarro et al., 2016a): the fundamental frequency *F_0_* of the plosives, the variations that this fundamental frequency presents in successive plosives, the irregular intervals that appear between plosives, the different vocalic sounds included (voiced versus unvoiced laughter), and very importantly the entropy, energy, and amplitude of the different frequencies that integrate the waveform. In the relationship between the sound structures of laughter and the linguistic utterances, it is very interesting that the entropy of the former appears to be higher than the entropy of the latter (Bea and Marijuán, 2003). Laughter is in general more disorganized, more “raucous,” and more energetic. One of the reasons is that the neuronal circuits in charge of laughter production are evolutionarily different, more primitive, than those involved in the production of spoken language (definitely, different neural systems converge in controlling the vowel cords, and the phonatory apparatus). It has also been argued that, in general, the increase of entropy in animal calls contributes to enhance their distinctiveness and attractiveness, improving the communication of emotional states between emitters and receivers (Takahashi et al., 2015). As we have already hinted (Navarro et al., 2016a), there might be a close interrelationship of sound entropies and neural entropies regarding the general “stuff” of brain processing (Friston, 2010; Carhart-Harris et al., 2014). Further up, the systemic repercussions of laughter, both physiological (respiratory, cardiovascular, immune, central nervous system, and autonomous nervous system, etc.) and molecular (release of dopamine, endorphins, neuropeptides, and general reward system, etc.), become essential to fulfill the informational missions of laughter in the synaptic consolidation of social bonds and to explain its multiple therapeutic applications in biomedical fields (Hasan and Hasan, 2009).

### Biomedical Applications of Laughter in Depression and in Mental Health

Therapeutic methods based on laughter have been widely applied in the prevention and treatment of major medical illnesses – relevant positive effects of both laughter and humor were authenticated in autoimmune pathologies, surgical recuperations, psychotherapeutic interventions, pain relief, general resilience, mental wellbeing, and patient empowerment, etc. See reviews by Hasan and Hasan (2009), Takeda et al. (2010), Gelkopf (2011), Ganz and Jacobs (2014), and Weems (2014). In the application of laughter to mental pathologies, however, the *discriminative potential* that laughter seems to contain has been left almost unexplored. The fact is the emission of laughter, and the reaction to it, are affected *differentially* within the major neuropsychiatric morbidities, such as schizophrenia, depression, psychoticism, and dementia, as well as in the neurodegenerative conditions. See reviews by Walter et al. (2006), Falkenberg et al. (2007), Uekermann et al. (2008), Takeda et al. (2010), and Ko and Youn (2011). A trait common to these mayor pathologies is the decrease in the social abilities of the patient in order to engage and participate of groupal processes and to successfully establish a new bonding relationship; which is also accompanied by a relative blocking of the hedonistic/reward systems in the individuals affected. Thereafter, laughter and the whole spontaneous responses to humorous stimuli become either severely restricted or conspicuously disorganized. The important point, as already stated, is that the *specificity* of these relevant laughter affections has not been explored yet. Subsequently, in the extent to which the specific mental conditions of individuals are reflected in the sound structures of laughter (Ruch and Heintz, 2014), an in-depth exploration and understanding of these structures could provide new intervention tools in mental-health fields – indeed providing a more ambitious horizon to the present therapeutic applications. As we discuss here, neurocomputational applications in diagnostics and prognosis, in the detection of healthy subjects versus patients, and in the assessment of recovery progresses, would not be too farfetched. As our previous works show (Navarro et al., 2014, 2016a,b) rather simple trials based on statistical analysis of laughter in depression patients can already be useful in those tasks.

Summing up, the software in charge of analyzing the sound variables, neural network classifiers in the present case, should perform an accurate discrimination between the laughter of patients and the laughter of controls so that it can be reliably used as an auxiliary tool to determine the diagnostic of depression and to gauge the severity of the disorder. In that regard, the results we will discuss look promising. Nevertheless, in the present application of artificial neural networks, as in many other biomedical instances (Esteva et al., 2017), the increase in computational power becomes crucial to achieve the pretended reliability of the diagnostic tool. Indeed artificial neural networks are opening a new window of observation on the amazing complexity of “biological computations” themselves. And laughter is amongst the most complex “computational” processes exhibited by human brains. It integrates the emotional and the cognitive, the individual and the social, the semantic and the pragmatic. In the present case, a deeper understanding of laughter, as the proposed methodology will try to demonstrate, would open up the possibility of designing a new kind of neuroprosthetics for the diagnosis and clinical follow-up of a variety of neuropsychiatric affections.

## Methodology

Two different aspects have to be considered: the experimental collection of laughter samples and the computational procedures. From the experimental point of view, the methodology followed to collect laughs is shown in Figure 1. As thoroughly described in Navarro et al. (2014, 2016a), it consists of the steps which follow.

### Participants

A total of 50 individuals participated in the study: 30 depression patients and 20 healthy controls, including women and men, with ages comprised in between 20 and 65. There were 20 women among the patients (66%) and 10 in the controls (50%). In order to estimate the patient severity, the four categories of the Hamilton Depression Rating Scale (HDRS) were used. Hard depression was found in 11 patients (36.7%), severe depression in 6 (20%), moderate depression in 10 (33.3%), and light depression was present in 3 patients (10%). A total of 934 laughs were registered, all of them recorded in response to humorous videos, and always watched in the company of a relative or a friend (to emphasize: laughter is always a socially directed signal). A couple of directional digital voice recorders provided the individual registers. We recruited more patients than controls so to count with representatives in all the categories of the classification of depression, and to be able to correlate these categories with their specific laughter registers. All participants were Spanish nationals, and not suffered any other mental disease or physical illness that prevented the realization of the study; all of them could follow the humor sketches of the videos and fill in the requested questionnaires. Further inclusion criteria were explained in Navarro et al. (2014). The Ethics Committee of Aragon revised and approved the clinical protocol of the study.

### Questionnaires

The severity of clinical depression symptoms was assessed by means of the Hamilton Depression Rating Scale (HDRS). The HDRS test, with the original 21-items, was used in its Spanish validated translation (Ramos-Brieva and Cordero-Villafafila, 1988). It is one of the most widely used scales to measure severity of depression and mood disorders, both in research and in clinical practice.

### Laughing Compilation

A number of humorous sketches and funny videos, collected from Internet, were selected by the author team and organized into sections. The videos provided sufficiently funny circumstances to evoke laughter in most kinds of people, without incurring in an excess of diversity. They mostly consisted of visual puns, cartoon sketches, ridiculous falls, verbal jokes, fragments of TV series, well-known movie characters, humorist performances, etc. A series of preparatory trials were set in order to organize the different sketches and to facilitate the generation of laughter. Finally the sketches were compiled in a 20 min session to be watched by the participants. During the recording sessions, the sounds of each participant were registered by means of a digital voice recorder, Olympus VN-712PC (Olympus Imaging Corp., Tokyo, Japan). The registers were made in a wav archive encoded in 16-Bit PCM format, sampled in the 50–10,000 Hz interval. In order to separate each laugh episode, a careful inspection was made both by hearing the recordings and by visualizing the waveforms obtained from the sound analysis program Adobe Audition. Specific software developed by the authors – a genuine “plosive automatic detector” – eliminated the environmental noises (e.g., from the video itself) and distinguished each laughter episode separately, so that the different laughter utterances could be analyzed, selected, and stored individually. In the evaluation of audio segments, for patients and controls, clarity was the main factor: exclamations and guttural noises were discarded, as well as overlapped speech–laugh and laugh–laugh segments. As a result of this meticulous inspection, we were making sure that the final laugh archives were recorded from only one participant, had well defined boundaries, and were free from interference noises such as exclamations, throat clearing, coughs, or humming – or else they would be eliminated. This partially manual process of evaluation was too complicated to be achieved entirely via software tools; at present the procedure is slow, but reliable enough (The future perspective is the development of programmable laughter detectors such as support vector machines and machine learning methods).

### Laughter Processing

In accordance with the mentioned convention (Urbain et al., 2013), laughter bouts contain a succession of discrete elements, or plosives, which appear as isolated energy peaks separated by silence intervals and repeated every 200–220 ms approximately – though not quite regularly. In order to analyze this wide range of acoustic shapes, segmentation in the time domain is required. Bouts appear, at the temporal domain, as a series of alternating maxima and minima within the envelope of the waveform amplitude. In this temporal context, the main features or variables which have been found most relevant in previous studies (Navarro et al., 2014, 2016a) for every plosive are:

• Time duration. For each one of the plosives, the temporal succession of beginnings and ends, counted in ms, provides an overview of the laughter episode and its rhythm structure.
• Fundamental frequency mean. It can be defined as the inverse of the smallest period of the vocal fold oscillation in the interval being analyzed (every 10 ms).
• First three formants. They correspond to spectral peaks in the whole range of the speech wave that provide information about the acoustic resonances in the vocal tract for every plosive.
• Average power or energy per sample. It is derived from the sound amplitude; it directly affects our perception of the fundamental frequency, what is called pitch. As a perception of the listener, pitch itself influences the subjective interpretation of sound energy.
• Shannon’s entropy. It can be defined as an entropic metric of the diversity or variable information contained in the patterns of a message. In laughter plosives, it refers to the variability of frequencies present in the waveform.
• Percentage of voiced/unvoiced signal. It represents the time that vibrating vocal cords spend over a plosive versus the silence interval with the next plosive.

These six variables were extracted for all the 934 well-formed laughs, each one obtained by following the selection criteria previously mentioned. In total, there were 517 laughs from patients and 417 from controls. It means an average of 17 laughs for patients and 21 for controls. The software performing the “plosive automatic detection” function was implemented in Matlab version R2014a. As a global outcome of this variable extraction, a data matrix comprising all plosives sorted by individual laugh archives was obtained, each one in a row, with the variable values located in columns. A seventh dichotomous variable was included about the subject’s health status (1, depression; 2, healthy or control).

### Computational Procedures: Artificial Neural Networks Training and Depression Detection

**FIGURE 2:**
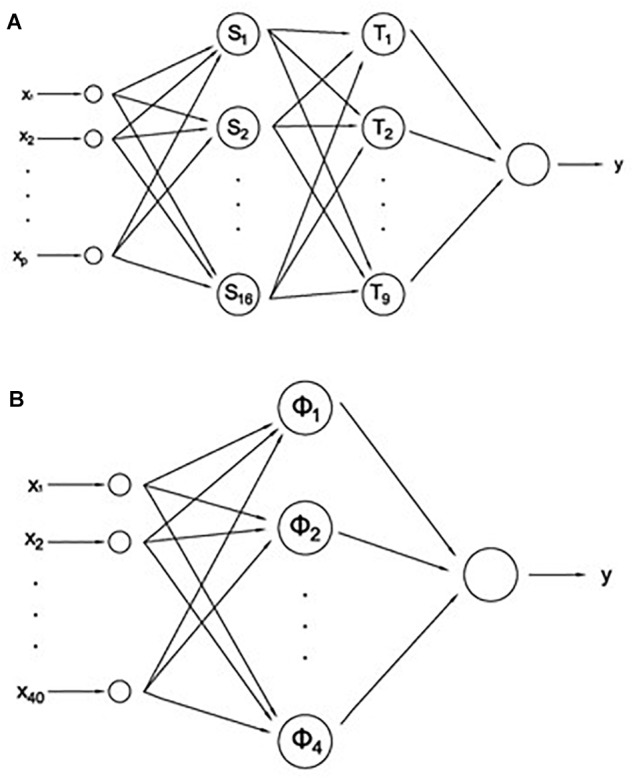
**(A)** Multilayer perceptron (MLP) neural network models: ANN (*p* = 40), EANN (*p* = 5), and 5PANN (*p* = 6). **(B)** Radial based function network (RBFN).

The present work improves the formal tools used in previous works (Navarro et al., 2014, 2016a,b) and also incorporates the empirical knowledge already gained on the relevance of the different variables. We have designed three models of multilayer perceptron (MLP) (Dayhoff, 1990) by means of the program Neurosolutions 5.0. In order to diagnose depression in a given subject through the MLP neural networks (Figure 2A), they were trained with static backpropagation according to a cross-validation method, using 80% of the data for training the neural networks (training data set) and the remaining 20% (testing data set) for evaluation of the network. We have designed three different models of MLP neural network. The models have been nominated ANN, EANN, and 5PANN. All the neural network models had two hidden or intermediate layers of 16 and 9 neurons. In the first hidden layer of 16 neurons, each neuron‘s transfer function was the hyperbolic tangent function, using backpropagation momentum learning rule with step size and momentum parameters equal to 1.0 and 0.7, respectively. The configuration of the second hidden layer of 9 neurons was identical in all characteristics to the preceding hidden layer except that the step size was set up to 0.1. The output layer in all neural networks was similar, with one output neuron in which the hyperbolic tangent function was defined as the activation function. The backpropagation momentum learning rule was defined with a step size equal to 0.01 and momentum 0.7.

The first of the networks, ANN, received as input the values of all predictive variables measured in the five plosives in each subject. Since 6 + 2 variables are measured in each plosive (including the patient/control distinction and the plosive placement, see section “Laughing Compilation”), the total number of input variables is equal to 40. Therefore, ANN classified the subjects by using all the available information about the laughter of individuals. The second network, EANN, received as input only the values of the energy, values measured in each one of the five plosives. This network classified the subjects by the tone of their laughter, which is the perceived pitch of a sound or the ear’s response to frequency. Finally, the third network, 5PANN, received as input only the predictor variables measured in the fifth plosive of each subject. In this case the neural network classified the subjects by the last laughter or fifth plosive, which by previous works we know is very predictive in the classification of subjects regarding their laughter.

In addition to the three MLPs we also studied the use of radial based function networks (RBFNs) for the recognition of depression out from subjects’ laughter (Figure 2B). RBFN (Castellanos et al., 2008) are hybrid networks which combine a RBF layer that uses Gaussian transfer functions with centers and widths set by unsupervised learning rules (e.g., Euclidean distance), and supervised learning (e.g., backpropagation) that is applied to the output layer. Three RBFNs, ANN, EANN, and 5PANN networks were built and tested, but only the first one obtained significant results. For this reason, in this work we included the two versions, MLP and RBFN, of the ANN network.

The RBFN version of ANN network was defined with an input layer with 40 neurons, as in its MLP counterpart, setting 8 clusters and using Euclidean distance. The hidden or intermediate layer had 4 neurons in which the hyperbolic tangent function was the activation function, and backpropagation momentum learning rule with step size and momentum equal to 1.0 and 0.7, respectively. Finally, the output layer consisted of a single neuron with activation function and backpropagation, with momentum learning rule parameters similar to the hidden layer except that the step size was equal to 0.1.

The training of the MLPs networks was carried out by setting a maximum number of epochs equal to 10,000 with MSE as the stop criterion. However, in the RBFN the maximum number of epochs during unsupervised and supervised training was equal to 100 and 10,000, respectively.

**FIGURE 3:**
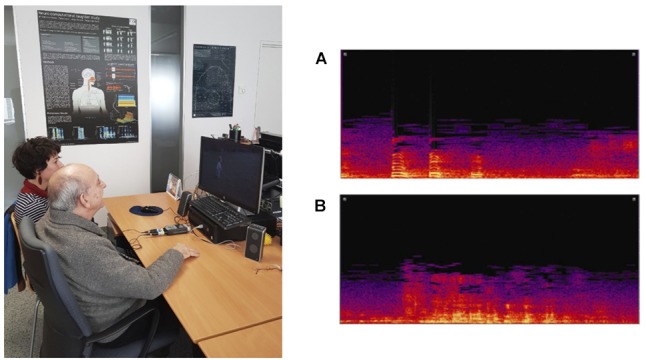
(Left) Experimental set-up. The prototype of the system is utilized with a double purpose. On the one hand the system is used for data collection, recording the laughter of patients with depression, and control subjects. On the other hand, and once the neural network is trained, the system is applied for the detection and diagnosis of depression. (Right) Sonogram of laughter recorded from a depressed patient **(A)**, and from a healthy or control subject **(B)**.

The different architectures were evaluated both for sensitivity analysis and for their performance in the classification of individuals, either as healthy subjects or patients with depression. In each of the neural networks we obtained the confusion matrix and, from the elements of the matrix, we calculated the accuracy, sensitivity, effectiveness, and precision of diagnosis for each one of the three networks. The whole results obtained, as will be discussed later, represent a neat improvement of this computational methodology and strengthen the capabilities of laughter to be used as a new auxiliary diagnostic tool (Figure 3). The sensitivity analysis consists of determining which input channels are most significant, and therefore what variables are important and have higher influence on the output of the network. By means of this analysis, the predictive value of each fundamental variable is estimated. This procedure eliminates those channels or input variables that are not significant, greatly reducing the network input and therefore its complexity.

Once a neural network was designed according to the data matrix (Figure 2), the training or learning process of the network was carried out. This required a different learning time for each neural network. And as a final step we obtained the weight matrix generated for each variable, as well as a series of performance measures for each artificial neural network: mean square error (MSE), standardized mean square error (NMSE), correlation coefficient (r), and error for global network accuracy (% error). In the study we also obtained two measures of the quality of the different neural network models, which will allow us to choose the neural network best suited to be used for practical purposes of diagnosis or classification: AIC (Akaike’s information criterion) and MDL (Rissanen’s minimum description length). AIC is used to select a neural network model adopting as criterion a compromise between the performance and the size of the network. The objective is to minimize this term as well as MDL, another measure for the selection of a model, in which the minimal value expresses the neural network model that optimizes the use of memory and maximizes success in prediction.

Finally, we obtained the ROC curve (Receiver Operating Characteristic) for each of the neural networks. The ROC curve is a plot for different cut-off settings of the sensitivity or proportion of true positives, i.e., subjects who really suffer from depression, against false positives, i.e., subjects diagnosed as depressed when they are healthy. In the present work this curve was used as a procedure to evaluate the reliability of the diagnosis provided by the neural network.

### Design of a Minimally Viable Prosthesis: Main Features

**FIGURE 4:**
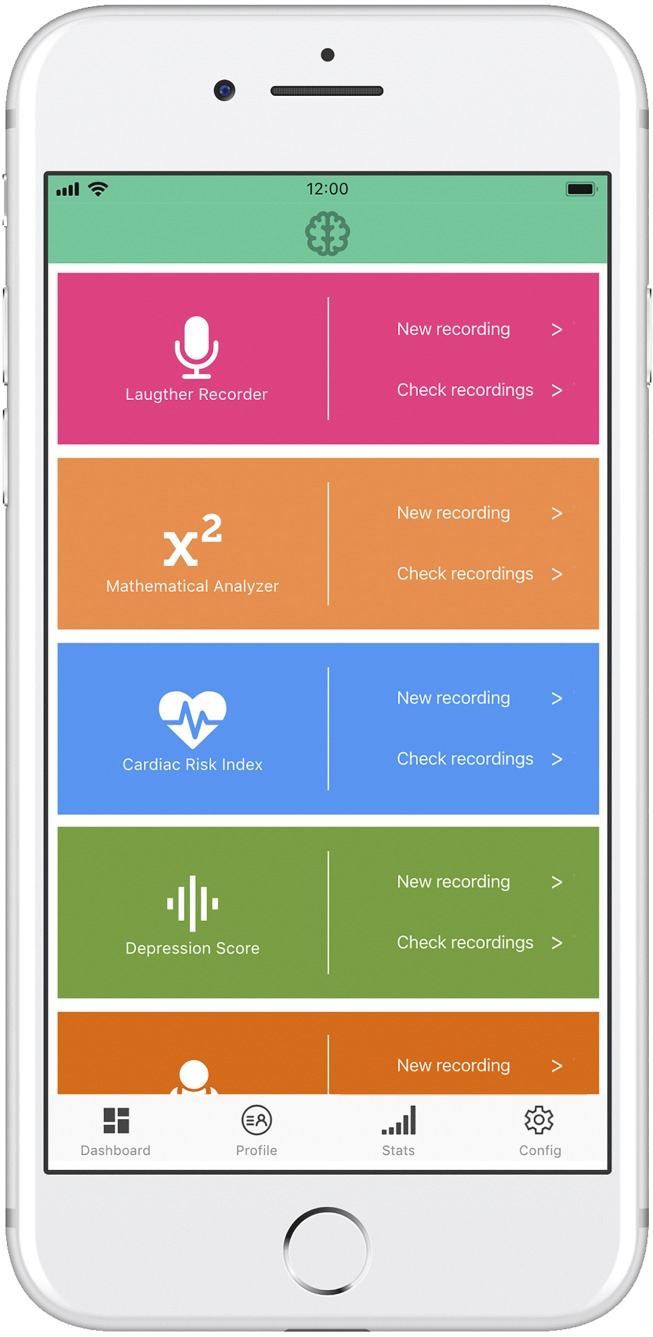
Prototype of a minimally viable neuroprosthesis implemented on a mobile phone. The depression detection module is accompanied by several clinical oriented modules, providing added value to the neuroprosthesis system.

A wearable laughter detector-analyzer (Figure 4) could incorporate the following functional modules:

(a) Receptor/recorder module, with capability to specifically detect the sounds of laughter versus the spoken language or other emotional utterances and physiological noises.
(b) Mathematical analyzer, with capability to extract the core variables identifying each laughter segment and its plosives.
(c) Neural network classifier, placing in the appropriate functional category the occurring laughter episodes.
(d) Output module, providing different kinds of specific and aggregate information on each episode, for instance, the “emotional” kind of laughter and its possible biomedical connotations (e.g., low activation and low entropy, potentially indicating the probability of depression; or the increased disorder and excess entropy accompanying neurodegenerative motor processes), as well as the daily and monthly laughing averages as useful indicators of general physical health and mental health.

Similar to the Smartwatch indicators used for fitness, the proposed laughter indicator could show a whole range of mental and physiological parameters related to, and influenced by, laughter quality and frequency.

## Results

The present work explores the possibility of using neural networks as an auxiliary tool in the clinical diagnosis of depression. The diagnosis is based on a set of descriptive variables, the values of which are derived from the quantitative analysis of spontaneous laughter. Thereupon, and according to the confusion matrices (Table 1), the sound variables chosen in the study should have sufficient predictive value to be used in the perceptron networks as input channel. As Table 1 indicates, individuals with depression are correctly classified by the three MLP networks (Figure 2A), ANN, EANN, and 5PANN, with a success rate of 91.15, 82.40, and 87.96%, respectively. Moreover, the RBF version (Figure 2B) of the ANN network (Table 4) correctly classifies 93.02% of subjects with depression, although this leads to a reduction of the percentage of success in the recognition of control subjects.

**Table 1:** Neural networks confusion matrices.

		Predicted depression (%)	Predicted controls (%)
ANN	Depression observed	91.15	8.84
	Controls observed	15.38	84.61
EANN	Depression observed	82.40	17.59
	Controls observed	14.45	85.54
5PANN	Depression observed	87.96	12.03
	Controls observed	6.02	93.97

In the sensitivity analysis (Tables 2, 5), i.e., the study of the predictive power of the different variables considered in the analysis of laughter, we obtained the following results. In the three MLP networks (Table 2), by rearranging the input variables in decreasing order of their predictive power, we realize that in the ANN network the best predictors are the entropy and time of the first plosive and the energy and time of the fifth plosive. With less relevance, there appear the entropy and the second formant of the fifth plosive. However, in the RBF model (Table 5) of the ANN network the relevant variables are entirely different. The most important variable regarding its predictive power is the entropy of the first plosive, following at a great distance the percentage of voiced/unvoiced signal in the second plosive and the second formant in the fourth and fifth plosives. In the case of the EANN network we obtained that the energy of the fifth plosive exhibits the maximum predictive power, followed by the energy of the first and fourth plosives, respectively. In particular, we found that in 5PANN the best predictors ranked in decreasing order of their predictive power were energy, entropy, and with a similar predictive power the first formant and the second formant.

**Table 2:** Validity analysis of neural networks as classifiers in depression detection.

	ANN	EANN	5PANN
Accuracy	0.87	0.83	0.90
Sensitivity	0.91	0.82	0.87
Effectiveness	0.84	0.85	0.93
Precision	0.85	0.85	0.93

In accordance with the above, we can conclude that the predictive power of the neural network and its worth as a tool in the diagnosis of depression based on laughter depends, as it should be expected, on the number of variables considered. Of all the variables entered as input in the three networks, *energy* and *entropy* were selected in terms of their predictive power as the two most important variables.

As for the analysis of validity of the MLP neural networks (Table 2) we observed that the maximum accuracy, effectiveness, and precision corresponds to 5PANN – the perceptron that only receives as input values the information of the fifth plosive. However the sensitivity, thus the proportion of individuals that are tested as depressed and are really depressed (True Positive), is maximum when ANN is used as classifier. Therefore, the neural network with 40 input channels is the best one at detecting depression. As Table 5 shows, in the RBF model of ANN a slight improvement in sensitivity is observed at the expense of accuracy, effectiveness, and precision. In a similar way, the analysis of the performance of the MLP as a classifier in the recognition of depression (Table 3) leads to the conclusion that the best classifier is the 5PANN network.

**Table 3:** Neural networks performance as classifiers in depression detection.

	ANN	EANN	5PANN
MSE	0.29	0.34	0.25
NMSE	0.38	0.43	0.31
R	0.78	0.75	0.82
%Error	13.68	17.71	12.31
AIC	1407.43	315.77	350.51
MDL	–	477.94	542.73

**Table 4:** RBF neural network confusion matrix.

	Predicted depression (%)	Predicted controls (%)
Depression observed	93.02	6.97
Controls observed	20.89	79.10

**Table 5:** Validity analysis of the RBF neural network as a classifier in depression detection.

	ANN
Accuracy	0.86
Sensitivity	0.93
Effectiveness	0.79
Precision	0.81

From a practical point of view, if an automatic diagnostic system had to be implemented, the selection criteria should be based on a compromise between the complexity of the neural network, the performance of the data during the training phase, and the number of cases. Following Table 3, and on this basis, the performance of MLP neural networks studied in the diagnosis of depression is established by comparing the AIC and MDL values. Since both values are lower in the 5PANN and EANN networks, we conclude that both models are preferable in the recognition of depression with respect to the ANN neural network. Moreover, the MDL value of these two neural networks is such that the use of memory is minimized and the success of the prediction is maximized. That is, using a 5PANN or EANN in a diagnostic problem, we will achieve a compromise between the complexity of the model (given by the number of variables) and the efficient use of data (the memory of the computer or machine where the neural network is implemented). This result is confirmed if we consider that the AIC value is also lower in the 5PANN and EANN networks, and subsequently the amount of information lost in these two perceptron models is lower with respect to the neural network ANN. It is interesting that if we compare the RBF version of the ANN network with the MLP models, the RBF neural network would appear, according to the AIC and MDL values (Table 6), in an intermediate position between the EANN, 5PANN, and ANN networks.

**Table 6:** RBF neural network performance as a classifier in depression detection.

	ANN
MSE	0.30
NMSE	0.37
r	0.79
%Error	15.19
AIC	555.27
MDL	745.39

**FIGURE 5:**
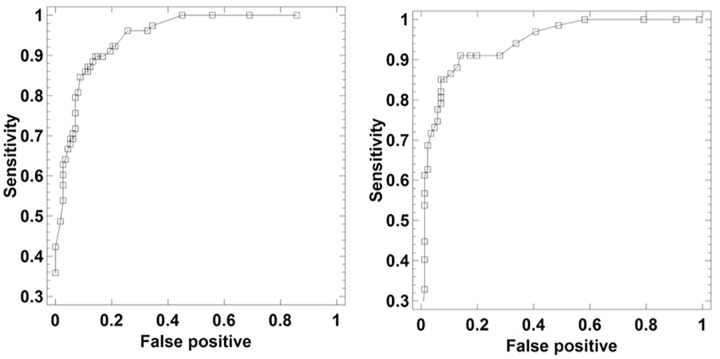
ROC curves of ANN (Left) and RBF (Right) neural networks as a tool for depression detection via laughter records.

**FIGURE 6:**
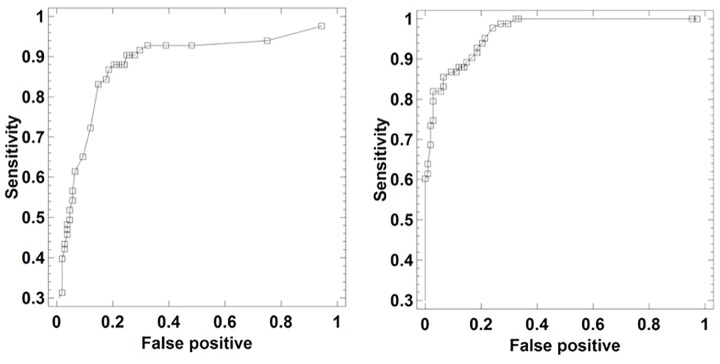
ROC curves of EANN (Left) and 5PANN (Right) neural networks as a tool for depression detection via laughter records.

Finally, the reliability analysis of the diagnosis with ANN (MLP and RBF models), 5PANN, and EANN networks was evaluated by obtaining the ROC curves (Diagnostic Performance Curves). The corresponding ROC curves (Figure 5, 6) are very close to the top left hand angle with appropriate values of sensitivity against false positive as displayed in Tables 2, 5.

## Discussion

Following the results obtained, neural network classifiers would perform the diagnosis of depression based on sound variables of laughter with a suitable level of precision. These results would open up the possibility of implementing a device or wearable laughter detector-analyzer as shown in Figure 4.

The results obtained show that the neural networks can be adequately trained at classifying subjects with depression by means of the quantitative analysis of laughter. When the network is subjected to training under cross-validation protocol, thus using 80% of the data to fit the weights and 20% for its evaluation, the success in the percentage of subjects with depression classified correctly exceeds 82%. These results are reflected in ROC curves that are characteristic of reliable diagnostic models, in which an appropriate classification of the subjects is obtained. The performance of the networks was similar to that obtained in previous studies (Navarro et al., 2014, 2016b) using multivariate statistical methods (e.g., discriminant analysis, j-biplot etc.) and data mining methods (e.g., decision trees, cluster analysis). A striking result is obtained with the sensitivity analysis. This analysis selects a subset of variables in the ANN neural network of the total available variables, this subset having sufficient predictive power to explain a precision of 91.15% in the diagnosis. Some of the selected variables are entropies in the first and fifth plosives as well as the energy in the fifth plosive. This result complies with the outcome of a previous study based only on entropies (Navarro et al., 2016a), demonstrating that depression could be diagnosed through decision trees exclusively based on entropies with 82.1% accuracy. Further, in the RBF model of the ANN network entropy appears again as a very relevant variable, this time in the first plosive and at a great distance of the other sound variables. This discriminative relevance of the entropy variable –and also of energy – tells us about the diminished information-processing capability of the depressed patients. They do not create sufficiently robust gradients of entropy (excitation/inhibition ratios) so that the automatic problem-solving mechanisms may get into action; and they cannot spontaneously elevate the “arch” of the fundamental frequency across the plosives either. The depressed laughter becomes weaker, less colored in frequencies, and flatter. From our point of view, this interpretation looks quite reasonable and fully expected, but without an adequate neurocomputational hypothesis, the whole phenomenon of the direct relationship between laughter and depression would pass unnoticed.

Comparing the results obtained with the other related works that have approached the detection of depression by means of speech analysis (Low et al., 2010; Alghowinem et al., 2013) we find that laughter performs slightly better than spontaneous language, both concerning single features or variables (e.g., energy, entropy, fundamental frequency) or their fusion. Most of the detection results in the tables of those two works contain values in the range of 70% (or even lower) and occasionally the 80% range. If we realize that laughter is a more direct and emotion-loaded signal, the slightly better performance of the models herein presented, in spite of their comparative lack of analytical sophistication, is not surprising. However, when the detection is performed via the correlation of different audiovisual behaviors (Sturim et al., 2011; Scherer et al., 2013), the results achieved by those works outperform the present ones, although considering the only audio modality they provide a lower accuracy. Indeed laughter has merits to be included within the new field, based on automatic analysis and machine learning methods, which is emerging devoted to the general recognition of depression, mood, and emotion (Valstar et al., 2016).

More specifically, discussing the formal tools utilized, classifiers such as Convolutional Neural Networks (He and Cao, 2018) as well as Gaussian Mixture Models and Support Vector Machines (Alghowinem et al., 2013) have proved useful in the detection of depression from the linguistic characteristics of the voice. But considering the lack of well-structured units in laughter (not comparable to syllables, words, and sentences) we do not know at this time whether Support Vector Machines (Schuller et al., 2007) and other machine learning classifiers would have the classification success of a MLP. Furthermore, in the Alghowinem et al. (2013) paper the success of the Gaussian Mixture Model and Support Vector Machine classifiers was a result of their hybridized application. Gaussian Mixture Models classifiers have been shown to be effective in detecting depression in adolescent subjects based on the prosodic (e.g., pitch and speech rate) and spectral characteristics of speech (Low et al., 2010). However, one of their disadvantages is the use of high-dimensional vector spaces (Sturim et al., 2011). Thus, the number of dimensions is considerably reduced with our protocol based on the use of perceptrons (e.g., EANN and 5PANN) when inputs are extracted from laughter.

An intriguing result obtained in the work of Navarro et al. (2014) showed that when the analysis of laughter is performed only in male patients, the percentage of patients correctly classified into one of the five categories of the Hamilton scale or HDRS (normal, minor depression, moderate depression, severe depression, and very severe depression) was 85.47%. But for women the percentage was only 66.17%. One partial explanation of this curious gender difference is that in males the fifth plosive is the most discriminating, while in females the five plosives studied would have the same weight or importance in diagnosis. Different potential causes of this phenomenon (which studies with larger population should either confirm or disproof) are discussed in Navarro et al. (2014). We believe that in the future it would be of interest, at least in the case of men, to design and implement artificial neural networks as auxiliary tools for the diagnosis of the *severity of depression* in accordance with the HDRS scale via laughter. Interestingly, a similar gender bias in the automatic detection of depression via linguistic items appears in one of the works already mentioned (Low et al., 2010).

Regarding further implications of laughter in biomedical fields, the present results buttress its possible use for the specific detection of neurodegenerative diseases too. An impressive array of new biomedical procedures are presently explored for the early diagnosis of neurodegenerative conditions: from the development of advanced biochemical probes and molecular detectors (e.g., “liquid biopsies” in blood), to EEG and neuroimaging patterns, to ocular-macular nanosecond laser exploration, to pupillometry, and to a number of procedures based on exercise and gait (non-linear) analysis on equilibrium platforms, cognitive and memory tests, linguistic performance, etc. (Uekermann et al., 2008; Danev and St Stoyanov, 2010; Van Orden et al., 2011; Stoessl, 2012; Grasso et al., 2014; Pievani et al., 2014). Laughter may well join that list of multifarious biomedical investigations. There are good reasons for that. Firstly, the intriguing emotional and cognitive characteristics inherent of laughter, with the ample swaths of brain cortical areas and medial and cerebellar regions involved, would represent a promising model-system of really high complexity regarding potential elements for advanced diagnostics. Thereafter, it seems plausible that conspicuous alterations appear in the relevant variables (entropy, energy, and *F*_0_) where most of the “neurocomputational code” of laughter is ensconced.

And secondly, there is another important characteristic of laughter, which has not explored here, that concerns its timing. In one sense, the non-standard placement of laughter relative to topic boundaries in linguistic exchanges may be indicative of failure to maintain engagement in dialogue, to achieve cognitive “closure” in the ongoing episode due to diminished processing capabilities (Bonin et al., 2014; Tanaka and Campbell, 2014). And in another sense, the timing of the plosives themselves (the excess of regularity as well as the uncoupling with respect to the decreasing airflow) may be indicative of motor disorganization. In sum, there would appear different, complementary ways to introduce the computational analysis of laughter in the early detection and prognosis of Alzheimer and Parkinson, which represent two really aggravated problems in contemporary healthcare systems. Although the specific decoding would not be easy, these two pathological conditions would presumably imprint their particular signature in a different way upon the whole variables and characteristics of laughter.

Needless to say that a series of further methodological improvements are needed in order to standardize the present detection procedures: from the contents of the video probes, to the plosive detection software, to the computational analysis itself, and to the social environment around the patient – the social nature of the laughter signal can never be forgotten. Notwithstanding the methodological difficulties ahead, advancing in the utilization of laughter as an auxiliary diagnostic and monitoring element could contribute to enliven and humanize the clinical ambience around the exploration of mental disorders. We should not forget either that laughter itself constitutes one of the most efficient natural procedures to mobilize neuroprotective molecular mechanisms contributing to physiological and mental wellbeing – enjoyment. As the authors witnessed during the recording sessions, patients welcomed and enjoyed the laughing experience of the study.

In sum, this work reinforces the plausibility of using artificial intelligence techniques, specifically artificial neural networks, as auxiliary neuroprosthetic tools for the diagnosis and monitoring of depression and (quite possibly) other mental health conditions, via the analysis of laughter records. The use of neuroprostheses for diagnosis may also be extended during the treatment and follow-up of the disease. In addition, including other devices into the same prosthesis could decrease the application of pharmacotherapy treatments and therefore reduce the dosage of drugs (adrenergic inhibitors, antipsychotics, stimulants, or serotonin and norepinephrine reuptake inhibitors, etc.). See Sanchez (2016). Indeed the use of neuroprosthetics in the treatment of mental disorders could open up exciting new therapeutic possibilities compared to current pharmacological and behavioral therapies. The main novelty of our procedure is that we use as input a very few sound variables of laughter that collectively seem to reflect rather accurately the mental state of the patient. We are looking for the short term application of such neuroprostheses in e-Health or telemedicine systems oriented to diagnosis and monitoring of patients with depression. Extending the procedure to the most relevant neurodegenerative pathologies, i.e., Alzheimer and Parkinson, would constitute the next step.

## Data Availability

The datasets for this study will not be made publicly available because of Data Protection Law.

## Ethics Statement

The protocol was approved by the Regional Ethics Committee of Aragon (CEICA), Acta number 11/2008.

## Author Contributions

PM and RL-B designed the study. JN and RdM recorded the laughter of patients and controls and made the laughter processing. RL-B, AC, and MF undertook the neural network analysis. JN and RdM wrote the first draft of the manuscript. RL-B and PM reviewed the manuscript and prepared the final version. All authors contributed to and have approved the present manuscript.

## Conflict of Interest Statement

The authors declare that the research was conducted in the absence of any commercial or financial relationships that could be construed as a potential conflict of interest. The handling Editor declared a shared affiliation, though no other collaboration, with several of the authors MF and RL-B.

## References

[B1] Alghowinem S. Goecke R. Wagner M. Epps J. Gedeon T. Breakspear M. (2013). “A comparative study of different classifiers for detecting depression from spontaneous speech,” in *Proceedings of the ICASSP, IEEE International Conference on Acoustics, Speech and Signal Processing*, (Piscataway, NJ: IEEE), 8022–8026. 10.1109/ICASSP.2013.6639227

[B2] Ariniello L. (2001). *Brain Briefings. Humor, Laughter and the Brain.* Washington, DC: Society for Neuroscience.

[B3] Bachorowski J. A. Owren M. J. (2002). “Vocal acoustics in emotional intelligence,” in *The Wisdom of Feelings: Psychological Processes in Emotional Intelligence*, eds Feldman Barrett L. Salovey P. (New York, NY: Cambridge University Press), 11–36.

[B4] Bachorowski J. Owren M. (2008). “Vocal expressions of emotion,” in *Handbook of Emotions*, eds Lewis M. Haviland-Jones J. Barrett L. (New York, NY: The Guilford Press), 196–210.

[B5] Bea J. A. Marijuán P. C. (2003). Informal patterns of laughter. *Entropy* 5 205–213. 10.3390/e5020205

[B6] Bonin F. Campbell N. Vogel C. (2014). Time for laughter. *Knowl. Based Syst.* 71 15–24. 10.1016/j.knosys.2014.04.031

[B7] Carhart-Harris R. Leech R. Hellyer P. Shanahan M. Feilding A. Tagliazucchi E. (2014). The entropic brain: a theory of conscious states informed by neuroimaging research with psychedelic drugs. *Front. Hum. Neurosci.* 8:20 10.3389/fnhum.2014.00020 PMC3909994 24550805

[B8] Castellanos A. Gonzalo R. Martinez A. (2008). Simultaneous control of chaotic systems using RBF networks. *Int. Book Ser. Inform.Sci. Comput.* 2 28–32.

[B9] Chattopadhyay S. Kaur P. Rabhi F. Rajendra Achaya U. (2012). Neural network approaches to grade adult depression. *J. Med. Syst.* 36 2803–2815. 10.1007/s10916-011-9759-1 21833604

[B10] Costa E Silva A. Ewbank Steffen R. (2017). The future of psychiatry: brain devices. *Metabolism* 69 S8–S12. 10.1016/j.metabol.2017.01.010 28162776

[B11] Danev S. I. St Stoyanov D. (2010). Early noninvasive diagnosis of neurodegenerative diseases. *Folia Med.* 52 5–13. 10.2478/v10153-010-0041-y 20836391

[B12] Dayhoff J. (1990). *Neural Network Architectures.* New York, NY: Van Nostrand Reinhold.

[B13] del Moral R. Navarro J. Lahoz-Beltra R. Bedia M. G. Serón F. J. Marijuán P. C. (2014). Cognitive and emotional contents of laughter: framing a new neurocomputational approach. *Int. J. Synth. Emot.* 5 31–54. 10.4018/ijse.2014070104

[B14] Devillers L. Vidrascu L. (2007). “Positive and negative emotional states behind the laughs in spontaneous spoken dialogs,” in *Proceedings of the Interdisciplinary Workshop on The Phonetics of Laughter*, Saarbrucken, 37–40.

[B15] Esteva A. Kuprel B. Novoa R. A. Ko J. Swetter S. M. Bla H. M. (2017). Dermatologist-level classification of skin cancer with deep neural networks. *Nature* 542 115–118. 10.1038/nature21056 28117445 PMC8382232

[B16] Falkenberg I. Kiángel K. Bartels M. Wild B. (2007). Sense of humor in patients with schizophrenia. *Schizophr. Res.* 95 259–261. 10.1016/j.schres.2007.06.006 17628438

[B17] Friston K. (2010). The free-energy principle: a unified brain theory? *Nat. Rev. Neurosci.* 11 127–138. 10.1038/nrn2787 20068583

[B18] Ganz F. Jacobs J. (2014). The effect of humor on elder mental and physical health. *Geriatr. Nurs.* 35 205–211. 10.1016/j.gerinurse.2014.01.005 24656050

[B19] Gelkopf M. (2011). The use of humor in serious mental illness: a review. *Evid. Based Complement. Altern. Med.* 2011:342837 10.1093/ecam/nep106 PMC3135316 19687190

[B20] Grasso M. Piscopo P. Confaloni A. Denti M. (2014). Circulating miRNAs as biomarkers for neurodegenerative disorders. *Molecules* 19 6891–6910. 10.3390/molecules19056891 24858274 PMC6271879

[B21] Hardt J. V. Kamiya J. (1978). Anxiety change through electroencephalographic alpha feedback seen only in high anxiety subjects. *Science* 201 79–81. 10.1126/science.663641 663641

[B22] Hasan H. Hasan T. F. (2009). Laugh yourself into a healthier person: a cross cultural analysis of the effects of varying levels of laughter on health. *Int. J. Med. Sci.* 6 200–211. 10.7150/ijms.6.200 19652724 PMC2719285

[B23] He L. Cao C. (2018). Automated depression analysis using convolutional neural networks from speech. *J. Biomed. Inform.* 83 103–111. 10.1016/j.jbi.2018.05.007 29852317

[B24] Hurley S. Dennett D. C. Adams R. B. Jr. (2011). *Inside Jokes: Using Humor to Reverse-Engineer the Mind.* Cambridge, MA: The MIT Press 10.7551/mitpress/9027.001.0001

[B25] Ko H. Youn C. (2011). Effects of laughter therapy on depression, cognition and sleep among the community-dwelling elderly. *Geriatr. Gerontol. Inter.* 11 267–274. 10.1111/j.1447-0594.2010.00680.x 21241447

[B26] Low L. S. A. Maddage N. C. Lech M. Sheeber L. Allen N. (2010). “Influence of acoustic low-level descriptors in the detection of clinical depression in adolescents,” in *Proceedings of the ICASSP, IEEE International Conference on Acoustics, Speech and Signal Processing*, (Piscataway, NJ: IEEE), 5154–5157. 10.1109/ICASSP.2010.5495018

[B27] Marijuán P. C. Navarro J. (2011). The bonds of laughter: a multidisciplinary inquiry into the information processes of human laughter^∗∗^. *arXiv*

[B28] Martin R. A. (2002). Is laughter the best medicine? Humor, laughter, and physical health. *Curr. Dir. Psychol. Sci.* 11 216–220. 10.1111/1467-8721.00204

[B29] Morreall J. (1987). *The Philosophy of Laughter and Humor.* Albany, NY: State University of New York Press.

[B30] Moxon K. A. Foffani G. (2015). Brain-machine interfaces beyond neuroprosthetics. *Neuron* 86 55–67. 10.1016/j.neuron.2015.03.036 25856486

[B31] Mukherjee S. Ashish K. Baran Hui N. Chattopadhyay S. (2014). Modeling depression data: feed forward neural network vs. radial basis function neural network. *Am. J. Biomed. Sci.* 6 166–174. 10.5099/aj140300166

[B32] Nair J. Nair S. S. Kashani J. H. Reid J. C. Mistry S. I. Vargas V. G. (1999). Analysis of the symptoms of depression – a neural network approach. *Psychiatr. Res.* 87 193–201. 10.1016/S0165-1781(99)00054-2 10579552

[B33] Navarro J. del Moral R. Alonso M. Loste P. Garcia-Campayo J. Lahoz-Beltra R. (2014). Validation of laughter for diagnosis and evaluation of depression. *J. Affect. Disord.* 160 43–49. 10.1016/j.jad.2014.02.035 24709021

[B34] Navarro J. del Moral R. Cuesta Alvaro P. Lahoz-Beltra R. Marijuán P. C. (2016a). The entropy of laughter: discriminative power of laughter’s entropy in the diagnosis of depression. *Entropy* 18 1–12. 10.3390/e18010036

[B35] Navarro J. del Moral R. Marijuán P. C. (2016b). Laughing bonds: a multidisciplinary inquiry into the social information processes of human laughter. *Kybernetes* 45 1292–1307. 10.1108/K-02-2016-0026

[B36] Pievani M. Filippini N. van den Heuvel M. Cappa S. Frisoni G. (2014). Brain connectivity in neurodegenerative diseases—from phenotype to proteinopathy. *Nat. Rev. Neurol.* 10 620–633. 10.1038/nrneurol.2014.178 25287597

[B37] Poeck K. (1985). “Pathological laughter and crying,” in *Handbook of Clinical Neurology*, Vol. I, ed. Frederick S. (Amsterdam: Elsevier), 219–225.

[B38] Provine R. R. (2000). *Laughter, A Scientific Investigation.* New York, NY: Viking.

[B39] Ramos-Brieva J. Cordero-Villafafila A. (1988). A new validation of the Hamilton rating scale for depression. *J. Psychiatr. Res.* 22 21–28. 10.1016/0022-3956(88)90024-6 3397906

[B40] Ruch W. F. Heintz S. (2014). Humour styles, personality and psychological well-being: What’s humour got to do with it? *Eur. J. Humour Res.* 1 1–24. 10.7592/EJHR2013.1.4.ruch

[B41] Sanchez J. C. (2016). *Neuroprosthetics. Principles and Applications.* Boca Raton, FL: Taylor and Francis Group.

[B42] Scherer S. Stratou G. Morency L. P. (2013). “Audiovisual behavior descriptors for depression,” in *Proceedings of ICMI’13*, (Pennsylvania Plaza, NY: ACM Press), 135–140. 10.1145/2522848.2522886

[B43] Schuller B. Batliner A. Seppi D. Steidl S. Vogt T. Wagner J. (2007). “The relevance of feature type for the automatic classification of emotional user states: low level descriptors and functionals,” in *Proceedings of the Interspeech*, (Antwerp: ISCA), 2253–2256.

[B44] Stoessl A. J. (2012). Neuroimaging in the early diagnosis of neurodegenerative disease. *Transl. Neurodegener.* 1:5 10.1186/2047-9158-1-5 PMC3506998 23211024

[B45] Sturim D. Torres-Carrasquillo P. Quatieri T. F. Malyska N. Mccree A. Ma L. (2011). “Automatic detection of depression in speech using gaussian mixture modeling with factor analysis,” in *Proceedings of Interspeech*, (Florence: ISCA), 2983–2986.

[B46] Szameitat D. P. Alter K. Szameitat A. J. Wildgruber D. Sterr A. Darwin C. J. (2009). Acoustic profiles of distinct emotional expressions in laughter. *J. Acoust. Soc. Am.* 126 354–366. 10.1121/1.3139899 19603892

[B47] Takahashi D. Y. Fenley A. R. Teramoto Y. Narayanan D. Z. Borjon J. I. Holmes P. (2015). The developmental dynamics of marmoset monkey vocal production. *Science* 349 734–738. 10.1126/science.aab1058 26273055

[B48] Takeda M. Hashimoto R. Kudo T. Okochi M. Tagami S. Morihara T. (2010). Laughter and humor as complementary and alternative medicines for dementia patients. *BMC Complement. Altern. Med.* 10:28 10.1186/1472-6882-10-28 PMC2896339 20565815

[B49] Tanaka H. Campbell N. (2014). Classification of social laughter in natural conversational speech. *Comput. Speech Lang.* 28 314–325. 10.1016/j.csl.2013.07.004

[B50] Uekermann J. Channon S. Lehmkámper C. Abdel-Hamid M. Vollmoeller W. Daum I. (2008). Executive function, mentalizing and humor in major depression. *J. Inter. Neuropsychol. Soc.* 14 55–62. 10.1017/S1355617708080016 18078531

[B51] Urbain J. Cakmak H. Dutoit T. (2013). “Automatic phonetic transcription of laughter and its application to laughter synthesis,” in *Proceedings of the Fifth Bian-Nual Humaine Association Conference on Affective Computing and Intelligent Interaction (ACII2013)*, Geneva, 153–158. 10.1109/ACII.2013.32

[B52] Valstar M. Gratch J. Ringeval F. Lalanne D. Torres M. T. Scherer S. (2016). AVEC 2016 – depression, mood, and emotion recognition workshop and challenge^∗∗^. *arXiv*

[B53] Van Orden G. C. Kloos H. Wallot S. (2011). “Living in the pink: intentionality, wellbeing, and complexity,” in *Philosophy of Complex Systems* 10 eds Hooker C. Gabbay D. M. Thagard P. Woods J. (Amsterdam: Elsevier), 629–672. 10.1016/B978-0-444-52076-0.50022-5

[B54] Walter M. Haonni B. Haug M. Amrhein I. Krebs-Roubicek E. Mäller-Spahn F. (2006). Humour therapy in patients with late-life depression or Alzheimer’s disease: a pilot study. *Int. J. Geriatr. Psychiatry* 22 77–83. 10.1002/gps.1658 16977676

[B55] Weems S. (2014). *Ha! The Science of When We Laugh and Why.* New York, NY: Basic Books.

